# DNA Methylation-Based Interferon Scores Associate With Sub-Phenotypes in Primary Sjögren’s Syndrome

**DOI:** 10.3389/fimmu.2021.702037

**Published:** 2021-07-16

**Authors:** Juliana Imgenberg-Kreuz, Johanna K. Sandling, Katrine Brække Norheim, Svein Joar Auglænd Johnsen, Roald Omdal, Ann-Christine Syvänen, Elisabet Svenungsson, Lars Rönnblom, Maija-Leena Eloranta, Gunnel Nordmark

**Affiliations:** ^1^ Section of Rheumatology and Science for Life Laboratory, Department of Medical Sciences, Uppsala University, Uppsala, Sweden; ^2^ Clinical Immunology Unit, Department of Internal Medicine, Stavanger University Hospital, Stavanger, Norway; ^3^ Molecular Medicine and Science for Life Laboratory, Department of Medical Sciences, Uppsala University, Uppsala, Sweden; ^4^ Rheumatology Unit, Department of Medicine Solna, Karolinska Institutet, Karolinska University Hospital, Stockholm, Sweden

**Keywords:** primary Sjögren’s syndrome, interferon, DNA methylation, autoimmunity, interferonopathies, precision medicine

## Abstract

Primary Sjögren’s syndrome (pSS) is an autoimmune inflammatory disease with profound clinical heterogeneity, where excessive activation of the type I interferon (IFN) system is considered one of the key mechanisms in disease pathogenesis. Here we present a DNA methylation-based IFN system activation score (DNAm IFN score) and investigate its potential associations with sub-phenotypes of pSS. The study comprised 100 Swedish patients with pSS and 587 Swedish controls. For replication, 48 patients with pSS from Stavanger, Norway, were included. IFN scores were calculated from DNA methylation levels at the IFN-induced genes RSAD2, IFIT1 and IFI44L. A high DNAm IFN score, defined as > mean_controls_ +2SD_controls_ (IFN score >4.4), was observed in 59% of pSS patients and in 4% of controls (p=1.3x10^-35^). Patients with a high DNAm IFN score were on average seven years younger at symptom onset (p=0.017) and at diagnosis (p=3x10^-3^). The DNAm IFN score levels were significantly higher in pSS positive for both SSA and SSB antibodies compared to SSA/SSB negative patients (p_discovery_=1.9x10^-8^, p_replication_=7.8x10^-4^). In patients positive for both SSA subtypes Ro52 and Ro60, an increased score was identified compared to single positive patients (p=0.022). Analyzing the discovery and replication cohorts together, elevated DNAm IFN scores were observed in pSS with hypergammaglobulinemia (p=2x10^-8^) and low C4 (p=1.5x10^-3^) compared to patients without these manifestations. Patients < 70 years with ongoing lymphoma at DNA sampling or lymphoma at follow-up (n=7), presented an increased DNAm IFN score compared to pSS without lymphoma (p=0.025). In conclusion, the DNAm-based IFN score is a promising alternative to mRNA-based scores for identification of patients with activation of the IFN system and may be applied for patient stratification guiding treatment decisions, monitoring and inclusion in clinical trials.

## Introduction

Primary Sjögren’s syndrome (pSS) is a chronic inflammatory disease with both organ-specific and systemic autoimmune manifestations (1). Focal lymphocytic infiltrates in the salivary and lachrymal glands are hallmarks of the organ-specific autoimmunity, causing the characteristic sicca symptoms. A proportion of patients displays signs of systemic autoimmunity. Autoantibodies against Sjögren´s syndrome antigen A (SSA)/Ro, subunits Ro52 and Ro60, and SSB/La, are present in sera from approximately 75% and 45% of patients, respectively. Hypergammaglobulinemia, leukopenia and extraglandular manifestations such as purpura or arthritis may be present (2). Patients with pSS have an increased risk of lymphoma, most commonly non-Hodgkin lymphomas of the B cell type arising in the mucosa associated lymphoid tissue (MALT) (3). The genetic background supports the clinical and serological heterogeneity of the disease, where only SSA/SSB antibody positive pSS present with an *HLA* association (1). This provides evidence for the distinction of two subgroups of pSS with different etiopathogenic backgrounds.

Activation of the type I interferon (IFN) system is regarded as one of the key mechanisms in the pathogenesis of pSS, most prominent in patients positive for SSA/SSB antibodies (4, 5). Immune complexes consisting of SSA/Ro and SSB/La proteins bound to nucleic acid and SSA/SSB antibodies ligate to endosomal toll-like receptor (TLR) 7. A signaling cascade is initiated, involving interleukin (IL)-1 receptor-associated kinase 4 (IRAK4), which leads to transcription of type I IFN genes (6). Type I IFNs bind to the ubiquitously expressed type I IFN receptor (IFNAR), activating the Janus kinase (JAK)/signal transducer and activator of transcription (STAT) pathway, ultimately leading to the transcription of thousands of IFN-induced genes, eliciting an immune response (7).

Direct measurement of IFN-α protein levels in sera has proven difficult due to the many type I IFN subtypes, the usually low concentration of IFNs under physiological conditions and the use of different methods. Instead, type I IFN system activation is commonly determined by assessing mRNA upregulation of a set of IFN-stimulated genes (ISGs), a so called “IFN signature”. An IFN score can be determined based on gene expression levels of a panel of ISGs in controls, calculated as sum of Z-scores (8). Approximately 55-80% of patients with pSS display an IFN signature in peripheral blood cells (9–12). As several biological treatments targeting the IFN system are currently under development or in clinical trials, sub-classification of patients with a high IFN system activation who could benefit particularly from IFN inhibition may be essential for future clinical trial outcomes. However, RNA is not always available and is subjected to degradation during long-term storage. DNA is more commonly collected and is more stable over time under most conditions. Recently, we have developed a method for assessing type I IFN system activation using DNA methylation (DNAm) data, which strongly correlates with mRNA-based IFN scores in multiple blood cell types (13). The aim of the current study was to determine potential associations between a DNAm-based IFN score and clinical and serological manifestations in patients with pSS.

## Material and Methods

### Patients and Controls

The discovery cohort consisted of 100 consecutive patients with pSS from the Rheumatology clinic at Uppsala University Hospital, Sweden. As control samples, 587 individuals were enrolled including 400 healthy blood donors from the Uppsala Bioresource (Uppsala University Hospital, Sweden) and 187 population controls from the Karolinska University Hospital (Stockholm, Sweden) (14). For replication, 48 patients with pSS from the Rheumatology unit at the Stavanger University Hospital, Norway were included. Clinical data presented ever during the disease course and treatment at DNA sampling were extracted from the medical records. Germinal center-like formations in minor salivary glands were assessed in hematoxylin and eosin staining with light microscopy as previously described (15). Autoantibody status was retrieved at the nearest time point to DNA sampling. All patients fulfilled the American Consensus Group (AECG) criteria for pSS, and all subjects provided informed consent to participate in the study. The study protocol was approved by the regional Ethics Boards.

### DNA Methylation-Based IFN Score

Genomic DNA was prepared from peripheral whole blood samples from patients with pSS and control individuals, and DNA methylation levels of 485,577 CpG sites were interrogated using the Illumina Infinium HumanMethylation450k BeadChip array as described previously (16, 17). Signal intensities were parsed into the R Minfi package for quality control and normalization, detailed in (16, 18). Methylation beta‐values were calculated as the fraction of the signal intensity from the methylated CpG sites over the total intensity (range 0 to 1, corresponding to 0 to 100% methylation). Relative distribution of major blood cell types in samples from controls and patients was estimated applying the method by Houseman et al., where publicly available reference DNA methylation profiles from flow sorted blood cell types are used to deduct the cell type composition of each sample included in the current study (19, 20). IFN system activation scores based on DNA methylation-beta levels at three CpG sites located at type I IFN regulated genes (cg05696877 at *IFI44L*, cg05552874 at *IFIT1*, and cg10549986 at *RSAD2*) were calculated according to the previously developed formula by Kirou et al. and described for DNAm data by Björk et al. (8, 11). Briefly, methylation-beta mean and standard deviation for each CpG site in the control group were used to achieve standardized values (Z-scores) for each individual according to the formula: Z-score = (value_individual_ – mean_controls_)/s.d._controls_. In the second step, Z-scores for the three CpG sites were summed up to total IFN scores. A high IFN score was defined as IFN score > 4.4, corresponding to DNAm IFN score > mean_controls_ +2 s.d._controls_. An IFN score ≤ 4.4 was defined as low IFN score.

### Statistical Analysis

Categorical variables were reported as number and percent, and continuous variables as mean and standard deviation. Correlations between two continuous variables were analyzed using Spearman’s rank correlation coefficient *ρ*. A two-tailed Mann-Whitney *U* test with continuity correction was applied to assess distributions of continuous variables between two groups. Kruskal-Wallis *H* with *post hoc* Mann-Whitney *U* test was used for comparisons between more than two groups. Frequencies between groups were assessed with X^2^-test or, for sparse data, with Fisher’s exact test. P-values < 0.05 were considered significant. Analyses were performed using R v.4.0.4 and GraphPad Prism v9, and graphs were prepared in R v.4.0.4 using ggplot2 with ggpubr and ggrides.

## Results

**Figure 1:**
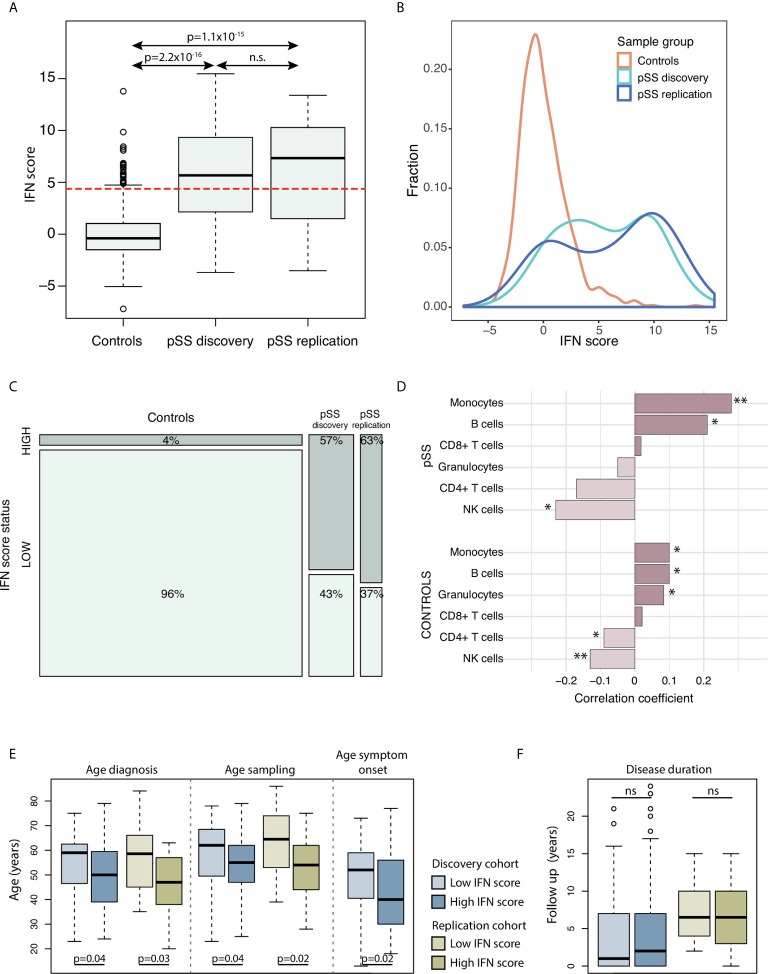
**(A)** DNAm IFN scores in controls (n=587), pSS discovery cohort (n=100) and pSS replication cohort (n=48). The dotted red horizontal line indicates the threshold for high IFN score (DNAm IFN score > 4.4 corresponding to > mean IFN score_ctrl_ +2s.d._ctrl_). **(B)** Density plot of IFN score distribution in controls (orange), pSS discovery cohort (light blue) and pSS replication cohort (dark blue). **(C)** Mosaic plot illustrating enrichment of high IFN score in the pSS discovery and pSS replication cohorts compared to controls. **(D)** Correlation between IFN score levels and fraction of major blood cell types in the pSS discovery cohort (upper part) and in controls (lower part). Bars with asterisks indicate significant correlations. **(E, F)** PSS patients in the discovery and replication cohorts stratified for IFN signature status (high *vs* low); for **(E)** age (in years) at diagnosis, DNA sampling, symptom onset, and **(F)** disease duration from diagnosis to DNA sampling in years. **(A)** Kruskal-Wallis with *post hoc* Mann-Whitney *U*. **(C)** X^2^-test. **(D)** Spearman’s rho. **(E, F)** Mann-Whitney *U*. *P < 0.05; **P < 0.01. Boxes indicate median and interquartile range, whiskers indicate total range.

The study included a total of 148 patients with pSS (discovery cohort n=100 from Uppsala, Sweden, and replication cohort n=48 from Stavanger, Norway) and 587 Swedish control individuals. Clinical data are presented in **Table 1**. IFN score levels in controls had by definition a mean of 0, and in our study material a standard deviation of ±2.2 and a median of –0.4 (range –7.2 to 13.8) (**Figures 1A, B**). The vast majority of controls (96%) presented a low IFN score, while a high IFN score was observed in 26 (4%) control individuals. The IFN score in the pSS discovery cohort varied between –3.7 and 15.5 with a mean of 5.6 ( ± 4.3) and a median of 5.7 (**Figures 1A, B**). In the replication cohort IFN scores between –3.5 to 13.4 were observed with a mean of 6.0 ( ± 4.9) and median of 7.3 (**Figures 1A, B**). Patients with pSS displayed a tendency for a bimodal distribution of the IFN score, with either a low IFN score ≤ 4.4, or a high IFN score around 10 (**Figure 1B**). There was a strong enrichment for IFN system activation in patients with pSS: A high IFN score, defined as IFN score > 4.4, was observed in 57 patients (57%) in the discovery cohort (p_discovery_=3.5x10^-30^) and in 30 patients (63%) in the replication cohort (p_replication_=1.8x10^-24^) compared with controls (**Figure 1C**). IFN score levels were similar between female and male patients with pSS (p_discovery_=0.567; p_replication_=0.335), as well as between female and male control individuals (p_controls_=0.365) (**Supplementary Figure S1**).

**Table 1:** Demographic and clinical characteristics of patients with primary Sjögren’s syndrome (pSS) included in the discovery cohort and the replication cohort.

	Discovery cohort (Uppsala, Sweden)	Replication cohort (Stavanger, Norway)	Total
Individuals, n	100	48	148
Age at DNA sampling, years, mean (±s.d.)	56.1 (±13.7)	56.7 (±13.7)	56.3 (±13.6)
Female, n (%)	89 (89%)	41 (85%)	130 (88%)
Autoantibody frequency^‡^, n/available (%)			
SSA	72/100 (72%)	38/48 (79%)	110/148 (74%)
Ro52	51/82 (62%)	n.a.	51/82 (62%)
Ro60	52/82 (63%)	n.a.	52/82 (63%)
SSB	41/100 (41%)	21/48 (44%)	62/148 (42%)
ANA	76/100 (76%)	41/48 (85%)	117/148 (79%)
Positive for any of the included autoantibodies	88/100 (88%)	43/48 (90%)	131/148 (89%)
DNAm IFN score			
IFN score, mean (±s.d.)	5.6 (±4.3)	6.0 (±4.9)	5.8 (±4.5)
High IFN score^§^, n (%)	57 (57%)	30 (63%)	87 (59%)
Clinical manifestations^¶^, n/available (%)			
Focus score, mean (±s.d, n available)	3.6 (±3.2, n=62)	2.5 (±2.8, n=48)**	3.1 (±3.1, n=110)
GC formation	11/51 (22%)	5/20 (25%)	16/71 (23%)
Leucopenia	33/98 (34%)	23/48 (48%)	56/146 (38%)
P-IgG > 15g/L	46/90 (51%)	19/37 (51%)	65/127 (51%)
C3 below normal limit	6/72 (8%)	7/47 (15%)	13/119 (11%)
C4 below normal limit	5/18 (28%)	7/45 (16%)	12/63 (19%)
Raynaud’s	33/99 (33%)	20/48 (42%)	53/147 (36%)
Arthritis	17/99 (17%)	4/48 (8%)	21/147 (14%)
Purpura	8/99 (8%)	2/48 (4%)	10/147 (7%)
Lymphadenopathy	22/99 (22%)	6/48 (13%)	28/147 (19%)
Hypothyroidism	24/99 (24%)	11/48 (23%)	35/147 (24%)
Lymphoma	14/100 (14%)	1/48 (2%)*	15/148 (10%)
Medication at DNA sampling, n (%)			
Prednisolon	7 (7%)	7 (15%)	14 (9%)
Antimalarials	14 (14%)	16 (33%)	30 (20%)
Immunosuppressants^†^	4 (4%)	1 (2%)	5 (3%)
Biologics^#^	0	1 (2%)	1 (0.7%)

^‡^Autoantibody status from medical records at nearest time point to DNA sampling.

^§^High IFN score defined as DNAm IFN score > mean_ctrl_ +2s.d._ctrl_, >4.4.

^¶^Clinical manifestations presented ever.

^†^Azathioprine (n=2), mycophenolate mofetil (n=1), chlorambucil (n=1), cyclophosphamide (n=1).

^#^Infliximab (n=1).

Frequencies between discovery cohort and replication cohort compared with X^2^-test or, if applicable, with Fisher’s exact test. Continuous variables between groups tested using Mann-Whitney U. *P < 0.05, **P < 0.01.

ANA, antinuclear antibodies; C3, complement component 3; C4, complement component 4; DNAm, DNA methylation; GC, germinal center; IFN, interferon; n.a., not available; s.d., standard deviation; SSA, anti-Sjögren’s syndrome antigen A antibodies (Ro52 and/or Ro60); SSB, anti-Sjögren’s syndrome antigen B antibodies (La).

### Cell Type Fractions

In order to study whether changes in the IFN score were related to the proportion of major blood leukocytes, we investigated potential correlations between IFN score and leukocyte fractions based on DNAm data. In patients with pSS, a positive correlation was observed for fractions of CD19^+^ B cells and monocytes (R=0.21, p_discovery_=0.039 and R=0.28, p_discovery_=6.6x10^-3^, respectively), while the fraction of NK cells was found to correlate negatively with the IFN score (R_discovery_=–0.23, p_discovery_=0.022) (**Figure 1D** and **Supplementary Figure S2A**). Fractions of CD4^+^ T cells, CD8^+^ T cells and granulocytes did not correlate with IFN score levels in patients with pSS. Accordingly, a positive correlation between fractions of B cells and monocytes, and a negative correlation of NK cells with the IFN score was also seen in control individuals (**Figure 1D** and **Supplementary Figure S2B**).

### IFN Score and Age

In patients with pSS, a tendency for negative correlation of the IFN score with age at sampling was noted (R_discovery_=–0.20, p_discovery_=0.0461; R_replication_=–0.19, p_replication_=0.193), while this trend was absent in control individuals (R_controls_=0.06, p_controls_=0.15) (**Supplementary Figure S3**). Stratification on IFN score status revealed that patients with a high IFN score were younger at pSS diagnosis (discovery: Δ_age_ =–5.4 years; replication: Δ_age_ =–9.7 years) and at DNA sampling for interrogation of DNAm (discovery Δ_age_ =–4.8 years; replication: Δ_age_ =–9.9 years) compared with pSS patients with a low IFN score (**Figure 1E**). For the discovery cohort, clinical data regarding age at symptom onset were available, where patients with a high IFN score on average were 6.9 years younger at symptom onset compared to patients with a low IFN score (p_discovery_=0.017) (**Figure 1E**). There was no difference in disease duration from diagnosis to DNA sampling between patients with high or low IFN scores (p_discovery_=0.60; p_replication_=0.85) (**Figure 1F**). **Table 2** shows the summarized results from the discovery and replication cohorts together.

**Table 2:** Association between DNA methylation-based IFN score status and clinical parameters and disease manifestations in patients with primary Sjögren’s syndrome (pSS) summarized for discovery and replication cohorts together.

Phenotype^‡^	Discovery and replication cohort together, n=148		
	High IFN score	Low IFN score	P-value
	**Mean (±s.d.)**	
Age at DNA sampling (years)	53.7 (±13.2)	60.1 (±13.6)	**2.5x10^-3^**
Age at diagnosis (years)	48.2 (±13.7)	55.0 (±12.8)	**3.0x10^-3^**
Age at symptom onset (years)	41.6 (±15.1)	48.5 (±14.6)	**0.017**
Disease duration (years)	5.8 (±6.1)	5.1 (±5.3)	0.61
Focus score	3.5 (±3.2)	2.8 (±2.9)	0.26
	**N/available (%)**		
Female sex	77/87 (89%)	53/61 (87%)	1
ANA	78/87 (90%)	39/61 (64%)	0.24
SSA	82/87 (94%)	28/61 (46%)	**0.012**
Ro52	39/47 (83%)	12/35 (34%)	**0.039**
Ro60	43/47 (91%)	9/35 (26%)	**4.2x10^-3^**
SSB	50/87 (57%)	12/61 (20%)	**4.0x10^-3^**
Positive for any of the included autoantibodies	86/87 (99%)	45/61 (74%)	0.29
Leucopenia (< 4.0x10^9^/L)	39/86 (45%)	17/60 (28%)	0.21
P-IgG > 15 g/L	52/74 (70%)	13/53 (25%)	**4.5x10^-3^**
C3 below normal limit	7/76 (9%)	6/43 (14%)	0.55
C4 below normal limit	12/42 (29%)	0/21 (0%)	**0.016**
GC formation in minor salivary glands	10/38 (26%)	6/33 (18%)	0.59
Raynaud’s phenomenon	28/86 (33%)	25/61 (41%)	0.58
Arthritis	13/86 (15%)	8/61 (13%)	0.95
Purpura	8/86 (9%)	2/61 (3%)	0.32
Lymphadenopathy	16/86 (19%)	12/61 (20%)	1
Hypothyroidism	21/86 (24%)	14/61 (23%)	1
Lymphoma, all	9/87 (10%)	6/61 (10%)	1

^‡^Clinical manifestations presented ever, with autoantibody status retrieved from time point nearest to DNA sampling.

IFN score status defined as high IFN score (>mean_ctrl_ +2s.d._ctrl_, >4.4) or low IFN score (≤4.4).

Patients with pSS were stratified on IFN score status (high versus low) and continuous variables between groups were tested using Mann-Whitney U. Categorical variables were assessed using X^2^-test, or for sparse data using Fisher’s exact test.

ANA, antinuclear antibodies; C3, complement component 3; C4, complement component 4; DNAm, DNA methylation; GC, germinal center; IFN, interferon; n.a., not available; s.d., standard deviation; SSA, anti-Sjögren’s syndrome antigen A antibodies (Ro52 and/or Ro60); SSB, anti-Sjögren’s syndrome antigen B antibodies (La).

Significant p-values in bold.

### Autoantibodies

**Figure 2:**
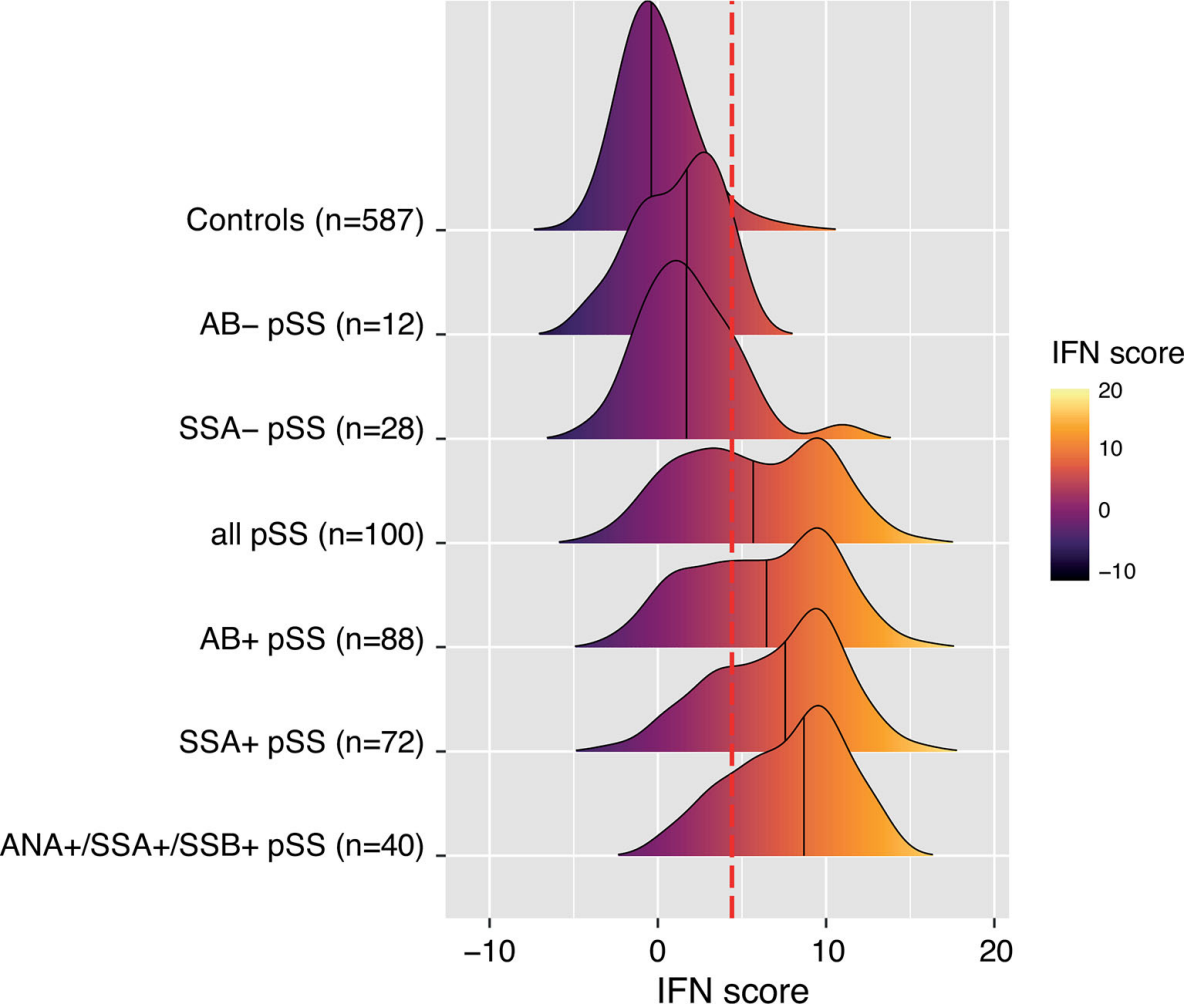
Ridges plot representing the distribution DNAm-based IFN scores in controls and subgroups of patients with pSS from the discovery cohort defined by autoantibody status. An individual patient can be part of one or several pSS subgroups. Ridges are sorted by increasing group median indicated by the black vertical line within each ridge. Ridges gradient indicates low (dark purple) to high (light yellow) DNAm IFN score levels. The dotted red vertical line indicates the threshold for high IFN score (DNAm IFN score > 4.4 corresponding to > mean IFN score_ctrl_ +2s.d._ctrl_).

**Figure 3:**
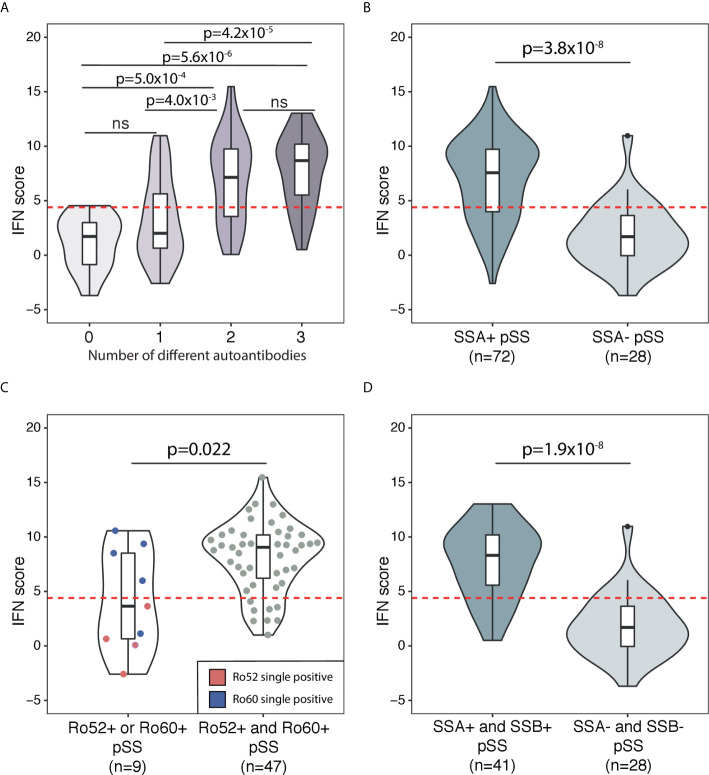
Association between DNAm IFN score levels and different autoantibody profiles in patients with pSS in the discovery cohort. The threshold for high IFN score is indicated by the dotted red horizontal line in each graph. Boxes within the violins indicate median and interquartile range, whiskers indicate total range. **(A)** Association between DNAm IFN score and number of different autoantibodies in sera from patients with pSS. Autoantibodies included in the analysis were ANA, SSA and SSB. **(B)** IFN score in SSA positive pSS compared with SSA negative pSS. **(C)** SSA positive pSS patients stratified for Ro52 or Ro60 single positivity (violin on the left with IFN scores of Ro52 single positive pSS indicated by red dots and Ro60 single positive pSS indicated by blue dots) compared with Ro52 and Ro60 double positive pSS (violin on the right with individual IFN scores of patients indicated by grey dots). **(D)** IFN score in SSA and SSB double positive pSS compared with SSA and SSB double negative pSS. **(A)** Kruskal-Wallis with *post hoc* Mann-Whitney *U*. **(B–D)** Mann-Whitney *U*.

Autoantibodies against ANA, SSA and SSB are prominent features of pSS in the majority of patients (21). In the current study, a total of 89% of patients expressed any of these autoantibodies in their sera (**Table 1**). Therefore, we sought to further dissect the association between the IFN score and autoantibody status. **Figure 2** depicts the distribution of the IFN score in controls and in overlapping groups of patients with pSS in the discovery cohort, displaying different autoantibody profiles. Patients with pSS positive for all three autoantibodies (ANA, SSA and SSB; n=40) showed the highest IFN score (mean 7.7 ± 3.6, median 8.7), while patients negative for all three autoantibodies (n=12) displayed an IFN score close to controls (mean 1.2 ± 2.5, median 1.7, p_discovery_=5.6x10^-6^) (**Figure 3A**). There was also a significant difference in IFN score levels between patients positive for all three autoantibodies compared to patients positive for only one of the antibodies (n=27, IFN score mean=3.4 ± 3.8, median 2.0, p_discovery_=4.2x10^-5^) (**Figure 3A**).

Previous studies have shown an upregulated IFN score on the mRNA level predominantly in patients with pSS positive for SSA/SSB antibodies (9, 10). In order to determine if the DNAm-based IFN score was also preferentially elevated in SSA/SSB antibody positive pSS, we dissected SSA/SSB antibody status further. In the discovery cohort, patients positive for SSA antibodies (n=72) showed a mean IFN score of 7.1 ± 3.8, while patients negative for SSA antibodies (n=28) had a mean IFN score of 1.8 ± 2.9 (p_discovery_=3.8x10^-8^) (**Figure 3B**). The association of SSA antibody positivity with IFN score elevation was confirmed in the replication cohort (SSA positive n=38 with IFN score mean=7.4 ± 4.2; SSA negative n=10 with IFN score mean 0.9 ± 3.5; p_replication_=4.8x10^-4^) (**Supplementary Figure S4**).

Information about SSA antibody subtype (Ro52 and/or Ro60) was available from 82 patients in the discovery cohort, (n=51 Ro52 positive and n=52 Ro60 positive, **Table 1**). Of these patients, nine (11%) were positive for only one of the SSA subtypes, while 47 (57%) were double positive. Single positivity for any of the SSA autoantibodies had an IFN score mean of 4.2 ± 4.7, while double positivity for both Ro52 and Ro60 was associated with a high IFN score mean of 8.1 ± 3.2 (p=0.022) (**Figure 3C**). In the discovery cohort, a total of 41 pSS patients were positive for autoantibodies against both SSA and SSB with a mean IFN score of 7.7 ± 3.3, compared with pSS negative for both SSA and SSB (n=28, mean IFN score 1.8 ± 2.9, p_discovery_=1.9x10^-8^) (**Figure 3D**). A significantly elevated IFN score in SSA and SSB positive compared to SSA and SSB negative pSS was confirmed in the replication cohort (p_replication_=7.8x10^-4^) (**Supplementary Figure S4**). Stratifying patents in the combined discovery and replication cohort for high (>4.4) or low (≤4.4) IFN score, confirmed the significantly higher frequencies of SSA/SSB antibodies in patients with a high IFN score (**Table 2**).

### Extraglandular Manifestations

**Figure 4:**
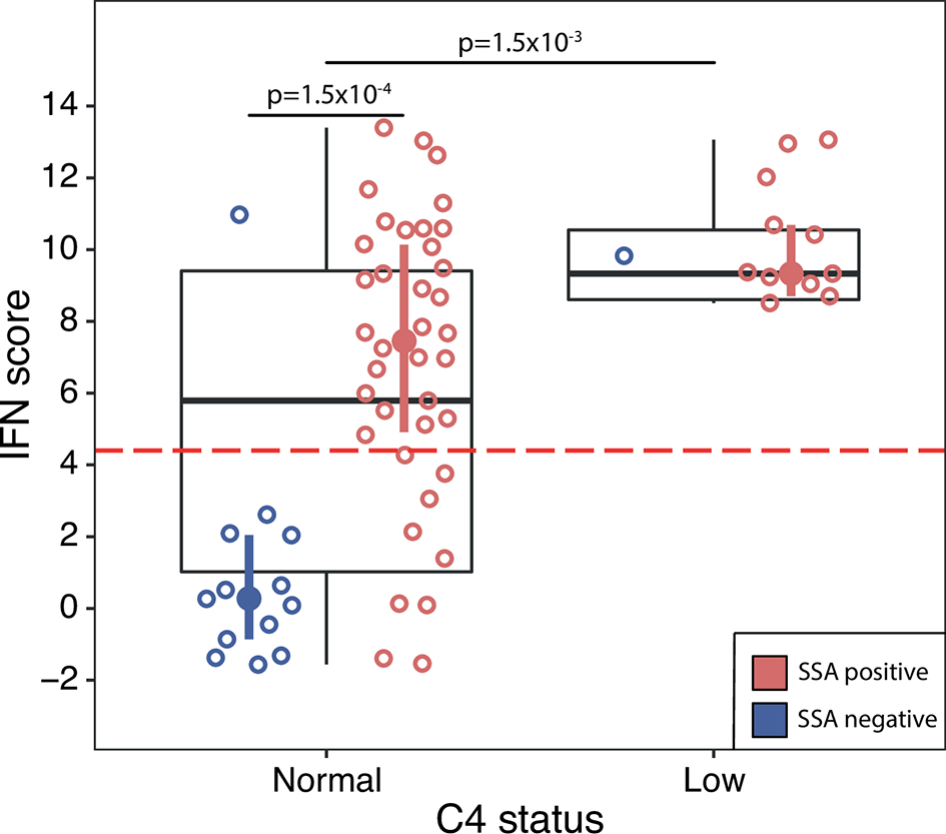
DNAm IFN score levels in pSS patients with normal C4 status (left box) compared with pSS patients with low C4 status (right box), discovery and replication cohort together. Dotted red horizontal line indicates threshold for high IFN score. Boxes represent median and interquartile range, whiskers indicate total range. Patients positive for SSA antibodies are indicated by red open circles, patients negative for SSA antibodies by blue open circles. IFN score median and interquartile range for pSS patients stratified on SSA status are indicated by red (SSA positive pSS) and blue (SSA negative pSS) filled circles and vertical lines, respectively. Differences between groups were assessed using Mann-Whitney *U*.

Next, we investigated if IFN score levels were associated with different laboratory or clinical manifestations present any time during the disease course. For these analyses the discovery and replication cohorts were analyzed together. We found that patients with leukopenia, hypergammaglobulinemia or low complement C4 displayed significantly higher IFN score levels compared to patients without these manifestations (**Table 3**). Stratifying patients into high or low IFN score revealed significantly higher frequencies of hypergammaglobulinemia and low C4 in patients with a high IFN score (**Table 2**). Further, we stratified patients’ C4 status on the presence of SSA antibodies. Among patients with normal C4 levels, this resulted in a bimodal distribution of IFN scores where patients positive for SSA antibodies had significantly higher IFN score levels (mean IFN score =7.0 ± 4.0) compared to patients negative for SSA antibodies (mean IFN score =1.1 ± 3.3, p=1.5x10^-4^) (**Figure 4**). All patients with low C4 levels had high IFN scores and all but one were SSA antibody positive. This patient was positive for RNP antibodies.

**Table 3:** Association between DNA methylation-based IFN score level and clinical manifestations of primary Sjögren’s syndrome (pSS).

Phenotype^‡^	Discovery and replication cohort together, n=148		
	DNAm IFN score, mean (±s.d.), n		P-value
	Positive phenotype	Negative phenotype
Leucopenia (< 4.0x10^9^/L)	7.0 (±4.6), n=56	5.0 (±4.2), n=90	**0.010**
P-IgG > 15 g/L	8.1 (±3.7), n=65	3.6 (±4.0), n=62	**2.0x10^-8^**
C3 below normal limit	5.1 (±5.9), n=13	6.3 (±4.1), n=106	0.41
C4 below normal limit	10.3 (±1.6), n=12	5.5 (±4.6), n=51	**1.5x10^-3^**
GC formation in minor salivary glands	5.9 (±3.8), n=16	5.2 (±4.5), n=55	0.60
Raynaud’s phenomenon	5.1 (±4.3), n=53	6.1 (±4.6), n=94	0.17
Arthritis	6.3 (±4.7), n=21	5.6 (±4.4), n=126	0.61
Purpura	6.6 (±3.7), n=10	5.7 (±4.5), n=137	0.63
Lymphadenopathy	5.7 (±4.2), n=28	5.7 (±4.5), n=119	0.95
Hypothyroidism	5.9 (±4.3), n=35	5.7 (±4.5), n=112	0.82
Lymphoma, all	6.3 (±5.5), n=15	5.7 (±4.3), n=133	0.62
Lymphoma onset at or after sampling, all (range 0-12 years)	7.7 (±5.3), n=9	5.7 (±4.3), n=133	0.20
Lymphoma onset at or after sampling, <70 years (range 0-12 years)	9.5 (±4.9), n=7	5.7 (±4.3), n=133	**0.025**
Lymphoma prior to sampling (range -5 to -19 years)	4.2 (±5.3), n=6	5.7 (±4.3), n=133	0.45

^‡^Clinical manifestations presented ever.

DNAm-based IFN score levels are reported as mean IFN score ±s.d. in patients with pSS stratified on their status for each of the disease manifestations included in the analysis. Continuous variables between groups were tested using Mann-Whitney U.

C3, complement component 3; C4, complement component 4; GC, germinal center; P-IgG, plasma immunoglobulin G.

Significant p-values in bold.

There were no differences in IFN score levels between patients with or without any extraglandular organ manifestations or the presence of germinal center-like formations in diagnostic minor salivary gland biopsies (**Table 3**). When comparing patients with a high or low IFN score, no difference between the patient groups was found for minor salivary gland histopathology or any extraglandular organ manifestation (**Table 2**). We stratified patients according to antimalarial (AMA) treatment or not. Of the 87 pSS patients with a high IFN score (>4.4), 20 (23%) were AMA treated at the time of DNA sampling. Of the 61 patients presenting with a low IFN score (≤4.4) 10 (16%) were treated with AMA (p=0.55). Conversely, AMA treated pSS had a mean DNAm IFN score of 6.5 ± 4.8, compared with non-AMA treated 5.6 ± 4.4, p=0.41.

### Lymphoma

**Figure 5:**
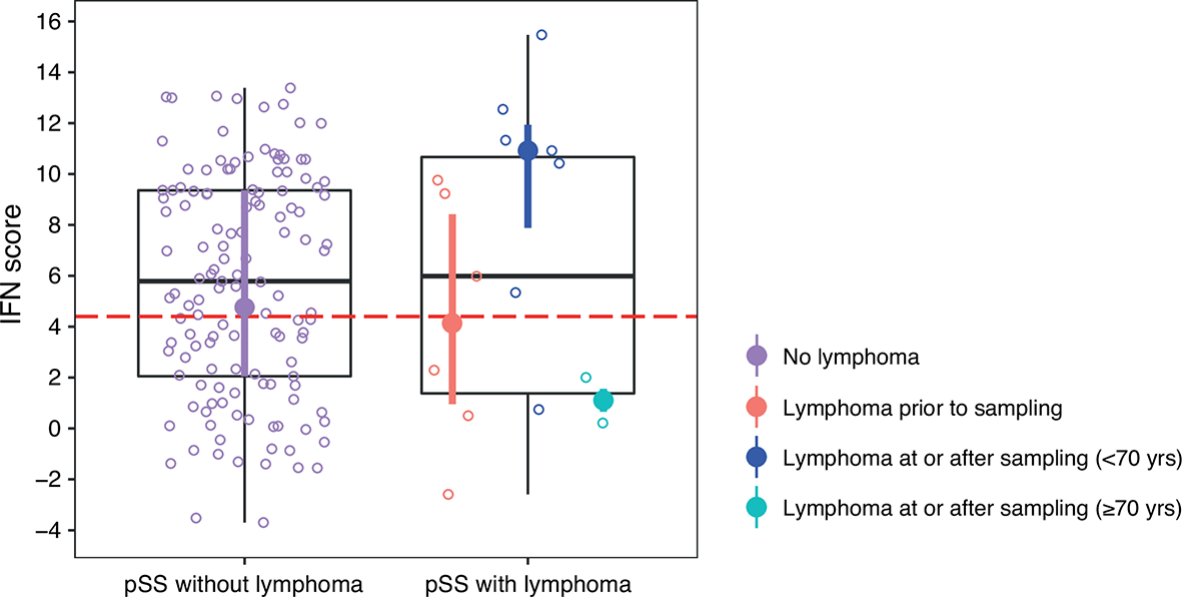
DNAm IFN score levels in patients with pSS stratified for lymphoma, discovery and replication cohort together. Dotted red horizontal line indicates threshold for high IFN score. Boxes represent median and interquartile range, whiskers indicate total range. pSS patients without lymphoma indicated in purple in the left panel. In the right panel pSS patients with lymphoma stratified for lymphoma prior to DNA sampling (in red), lymphoma at or after DNA sampling in patients < 70 years of age at lymphoma diagnosis (in dark blue), and lymphoma at or after DNA sampling in patients ≥ 70 years of age at lymphoma diagnosis (in light blue). DNAm IFN score median and interquartile range for each of the pSS subgroups are indicated by filled circles and vertical lines colored according to the corresponding patient subgroup.

There were 15 patients with lymphoma in the current study out of which eight (53%) presented with MALT lymphoma. Six patients had been diagnosed with lymphoma prior to DNA sampling (range –19 to –5 years) and nine patients were diagnosed with lymphoma concomitantly with DNA sampling ( ± 2 months, n=3) or at follow-up (range 1 to 12 years, n=6) (**Supplementary Table S1**). Of the six patients with prior lymphoma, two had received chemotherapy (13 and 5 years before DNA sampling, respectively) and four patients had received surgery (in three cases plus radiation). There was no difference in IFN score levels between patients with or without lymphoma, or between patients with ongoing and lymphoma at follow-up compared with patients without lymphoma (**Table 3** and **Figure 5**). Two SSA and SSB antibody negative patients developed non-MALT lymphomas at follow-up, aged >70 years (73 and 82, respectively). As old age in general is a risk factor for lymphoma and the antibody profiles and lymphoma subtypes were not typical for pSS, we excluded those two patients from the analysis (22). Six out of seven remaining patients with ongoing or follow-up lymphomas displayed a high IFN score, and the association between elevated IFN score levels (mean IFN score =9.5 ± 4.9) compared to pSS without lymphoma (mean IFN score = 5.7 ± 4.3) was strengthened (p=0.025) (**Table 3** and **Figure 5**). The low total number of lymphomas in the study precluded any analyses stratified for different lymphoma subtypes.

## Discussion

This is the first study using a DNAm-based IFN score in whole blood to assess clinical sub-phenotypes in any systemic inflammatory autoimmune disease. We found a high IFN score in 59% of patients with pSS compared with only 4% of controls. This is in concordance with previous studies using mRNA-based IFN signatures (9–12). Further, we demonstrate an association between IFN score levels and the presence of SSA and SSB antibodies, and a particularly elevated IFN score in patients displaying both SSA/Ro52 and SSA/Ro60 antibody subtypes. Interestingly, a recent study by Armağan et al. investigated clinical and serological profiles of patients with pSS being single or double positive for Ro52 and Ro60 antibodies (23). They found a higher prevalence of markers for B cell activity in patients double positive for Ro52 and Ro60 antibodies compared with single positive patients. The results from our study corroborate the findings of an activated immune system also with IFN system activation, being most prominent in patients double positive for Ro52 and Ro60 antibodies.

We found that patients presenting with hypergammaglobulinemia or low C4 during their disease course, displayed higher IFN score levels compared to patients negative for these phenotypes. Stratifying patients into high and low IFN score status confirmed the increased frequency of high IgG and low C4 in patients with a high IFN score. Previous studies using mRNA-based type I IFN scores have consistently found a higher frequency of SSA/SSB antibody positivity, hypergammaglobulinemia and lymphopenia in IFN score positive patients (9–11, 24). Furthermore, associations between a positive IFN score and rheumatoid factor (RF), low complement C3 or C4 and neutropenia has been described (9, 11).

A proposed mechanism for the interaction between IFN system activation and B cell activity are the SSA/SSB containing immune complexes, activating both complement and stimulating IFN gene transcription. Interaction between B cells and the professional IFN-producing plasmacytoid dendritic cells (pDCs) results in increased immune complex mediated type I IFN production (25). Further, the interaction between pDCs and immune complexes drives the expansion of peripheral B cell subsets (26). Type I IFNs stimulate different immune cells including B cells with induction of plasmablast differentiation, immunoglobulin production and further SSA/SSB antibody synthesis (27). This will perpetuate a vicious circle with IFN system and B cell activation in patients with pSS positive for SSA/SSB antibodies (28). Our finding that all patients with low C4 levels were SSA or RNP antibody positive supports this notion.

Patients with leukopenia during their disease course had higher IFN score levels compared to patients without leukopenia. Leukopenia has been associated with increased IFN-α levels and IFN-α-inducing capacity of sera from patients with SLE (29). One possible mechanism is that circulating IFN-α can lead to increased adhesion of leukocytes to the vessel. The mRNA-based IFN signature has not been associated with leukopenia *per se*, but with lymphopenia and neutropenia (9, 10). In contrast, we observed a positive correlation between the IFN score and the fraction of monocytes and B cells. Both monocytes and B cells are IFN-producing cells that also express the IFNAR, which amplifies the IFN response in a positive feedback manner (30, 31). We noted a weak negative correlation between the IFN score and the fraction of NK cells. The role of NK cells in pSS pathogenesis is unclear, and reduced NK cell numbers and activity in pSS have been reported, as well as a shift within the NK cell sub-populations (32, 33). The reasons behind the negative correlation observed is unclear and needs to be further explored.

While previous mRNA-based IFN scores studies have determined the association between IFN score positivity and laboratory parameters, the association with clinical manifestations is less clear. A weak association between an IFN signature and the cutaneous, hematological or biological domains of the EULAR Sjögren´s syndrome disease activity index (ESSDAI) has been reported, whereas Bodewes et al. only found an association with the biological domain (includes cryoglobulinemia, low complement, hypergammaglobulinemia or clonal component) (9, 10, 34). Activity in other extraglandular organs have not differed between IFN high and low patients. ESSDAI scores from the time of DNA sampling were not available from our patients, but associations with the cumulative extraglandular manifestations were not detected. We conclude that IFN system activation predominantly affects serological markers with less impact on clinical manifestations in pSS. The reason for this is unclear, but it is possible that other immunopathological mechanisms are of importance for certain clinical manifestations.

One exception might be the development of lymphoma. We hypothesized that patients with either ongoing lymphoma or lymphoma at follow-up may be more likely to present with a high IFN score as a sign of immune system activation. Our results indicate high IFN score levels as a possible predictor for lymphoma development, but the findings need to be confirmed in larger cohorts of patients with lymphomas of different subtypes. *Nezos* and co-workers did not find any difference in type I IFN score in peripheral blood between pSS lymphoma and pSS non-lymphoma patients (11). However, in MSG biopsies, pSS-lymphoma patients showed a predominance of type II IFN and a significantly increased IFN-γ/IFN-α ratio compared with pSS non-lymphoma. Further longitudinal studies of the impact of IFNs in peripheral blood or diagnostic MSG biopsies for lymphomagenesis are needed.

Patients with an earlier disease onset and younger age at DNA sampling, presented with higher IFN score levels. Younger patients are more often SSA/SSB antibody positive and present with systemic features of pSS, which might explain this association. Men with pSS often have a more active disease with pulmonary involvement and a higher frequency of lymphomas (35, 36). There were no differences in IFN score between male and female patients in this study where only 16 males were included. Larger studies including more male patients are warranted before conclusions regarding possible sex differences in IFN system activity can be drawn. Since AMA treatment has been shown to impair type I IFN activation, one might have expected a lower DNAm IFN score in AMA-treated patients with pSS (12, 37, 38). It can be speculated that AMA treatment would be particularly common in patients with active disease and presumably stronger activation of the IFN system, and that the IFN score in these patients might have been even higher prior to AMA treatment.

Direct measurement of type I IFNs has been proven challenging. Therefore, IFN scores based on mRNA expression of ISGs are commonly used as a proxy. Recently, we have developed alternative tools using DNAm or protein expression that allow the assessment of IFN activation under circumstances where RNA material is missing (13). We found in general a high correlation with the IFN score derived from mRNA expression in monocytes and B cells. Instead of using genome-wide methylation arrays, for practical reasons the DNAm IFN score might be generated in a more time- and cost-effective manner by interrogation of DNAm levels in a small panel of CpG sites by e.g. pyrosequencing or qPCR of bisulfite-converted DNA. It can be speculated whether IFN scores based on DNAm data may be more stable over time compared to mRNA or protein expression-based scores, and therefore more suitable for patient classification. On the other hand, mRNA expression-based scores may be more prone to dynamic changes in response to environmental and/or endogenous stimuli, and therefore more informative as biomarkers e.g. for monitoring disease activity and treatment responses. Studies simultaneously assessing IFN scores derived from gene expression, protein expression and from DNAm data in samples collected at different time points during the disease course are needed to further disentangle their relation and functional implication in pSS.

Several biological treatments targeting the IFN system are currently under development. Trials targeting B cells by blocking with anti-CD20 or anti-BAFF antibodies have shown an effect on biological markers, but less efficacy on most clinical manifestations (39, 40). Chloroquine interferes with endosomal TLR-signaling and inhibits plasma cell differentiation and class switch, but had no effect on symptoms of dryness, pain and fatigue in a previous study (38, 41). Potential therapeutics within the IFN system are antibodies against IFN-α, the IFNAR1 subunit (Anifrolumab) or molecules targeting JAK1, TLR7 or IRAK4 (42, 43). Sub-classification of patients who present with a high IFN system activation score and therefore could benefit particularly from IFN inhibition may be essential for future clinical trial outcomes.

The strengths of this study are the well characterized pSS patients from two Scandinavian cohorts of homogenous genetic background. The results from the discovery cohort are consistently replicated, confirming their validity. DNAm data in conjunction with clinical phenotype data have not been presented before. This study largely confirms the results from the mRNA-based IFN score, i.e. the strong association between IFN system activity and the presence of SSA antibodies. The concordance between mRNA-based IFN score and DNAm-based IFN score justifies the use of DNAm-based IFN scores when RNA is not available. Limitations are the low frequencies of certain clinical manifestations such as interstitial lung disease, which precluded any analyses, the low frequency of lymphomas, the low number of male patients in the study and the lack of ESSDAI at the time of DNA sampling. Since our study is limited to Scandinavian patients with pSS, similar investigations in multi-ethnic cohorts are required for studying generalizability of the results across different ancestries.

## Conclusion

PSS is a heterogeneous disease with an apparent need for biomarkers to identify patient subgroups for monitoring, prognosis, treatment and inclusion in clinical trials. Further analysis of patient sub-phenotypes may advance our understanding of the underlying molecular mechanisms of the disease and add novel insights in the etiopathogenesis. The results of this study suggest that activation of the type I IFN system in patients with pSS is mainly driven by SSA antibody positivity. The DNAm-based IFN score is a reliable alternative to mRNA-based scores when RNA material is not available.

## Data Availability Statement

Raw or normalised data supporting the conclusions of this article are available upon request from the authors.

## Ethics Statement

The studies involving human participants were reviewed and approved by Regional Ethics boards in Uppsala, Stockholm and Western Norway. The patients/participants provided their written informed consent to participate in this study.

## Author Contributions

JI-K and GN conceived the study. GN, LR, KN, SJ, RO, ES, and M-LE collected patient and control material and clinical data. JI-K, JS, GN, and A-CS were responsible for analysis of DNA methylation. JI-K analyzed the data and wrote the manuscript together with GN and M-LE. All authors contributed to the article and approved the submitted version.

## Funding

This study was supported by grants from the Knut and Alice Wallenberg Foundation (KAW 2011.0073), the Swedish Research Council for Medicine and Health (VR-MH Dnr 521-2014-2263 to A-CS, Dnr 2018-02399 to LR, and Dnr 2016-01982 to GN), the Swedish Rheumatism Association (to GN, M-LE, and LR), the King Gustaf V’s 80-year Foundation (to GN), the Swedish Society of Medicine (to JI-K), Selander Foundation (to JI-K), Marcus Borgström Foundation (to J-IK), the Western Norway Regional Health Authority and the Ingegerd Johansson Donation. The SNP&SEQ Technology Platform that performed the DNA methylation analyses was supported by Science for Life Laboratory, Uppsala University, the Knut and Alice Wallenberg Foundation and the Swedish Research Council (VR-RFI).

## Conflict of Interest

The authors declare that the research was conducted in the absence of any commercial or financial relationships that could be construed as a potential conflict of interest.

## Supplementary Material

Supplementary Figure 1 DNAm IFN score levels in controls, pSS discovery cohort and pSS replication cohort stratified for sex. Boxes represent median and interquartile range, whiskers indicate total range. Associations were tested using Mann-Whitney *U*. F, female; M, male.

Supplementary Figure 2 Correlation between DNAm IFN score and fractions of major blood cell types (CD4+ T cells, CD8+ T cells, NK cells, CD19+ B cells, monocytes, and granulocytes) in samples from **(A)** patients with pSS and **(B)** controls. Correlations were assessed using Spearman’s rho.

Supplementary Figure 3 Correlation between DNAm IFN score and age at DNA sampling (in years) in **(A)** patients with pSS in the discovery cohort, **(B)** patients with pSS in the replication cohort and **(C)** controls. Correlations were assessed using Spearman’s rho.

Supplementary Figure 4 Association between DNAm IFN score levels and different autoantibody profiles in patients with pSS in the replication cohort. Threshold for high IFN score is indicated by the dotted red horizontal line in each graph. Boxes within the violins indicate median and interquartile range, whiskers indicate total range. **(A)** Association between DNAm IFN score and number of different autoantibodies in sera from patients with pSS. Autoantibodies included in the analysis were ANA, SSA and SSB. **(B)** IFN score in SSA positive pSS compared with SSA negative pSS. **(C)** IFN score in SSA and SSB double positive pSS compared with SSA and SSB double negative pSS. **(A)** Kruskal-Wallis with *post hoc* Mann-Whitney *U*. **(B, C)** Mann-Whitney *U*.

Supplementary Table 1 Patients with primary Sjögren’s syndrome and lymphoma.
