# Supplementary material for: DNA Methylation-Based Interferon Scores Associate With Sub-Phenotypes in Primary Sjögren’s Syndrome
Source: Front Immunol. 2021 Jul 16;12:702037. doi: 10.3389/fimmu.2021.702037 (PMC8322981; doi:10.3389/fimmu.2021.702037)
Supplement: Supplementary Figure 1 — DNAm IFN score levels in controls, pSS discovery cohort and pSS replication cohort stratified for sex. Boxes represent median and interquartile range, whiskers indicate total range. Associations were tested using Mann-Whitney U. F, female; M, male. [file DataSheet_1.pdf]

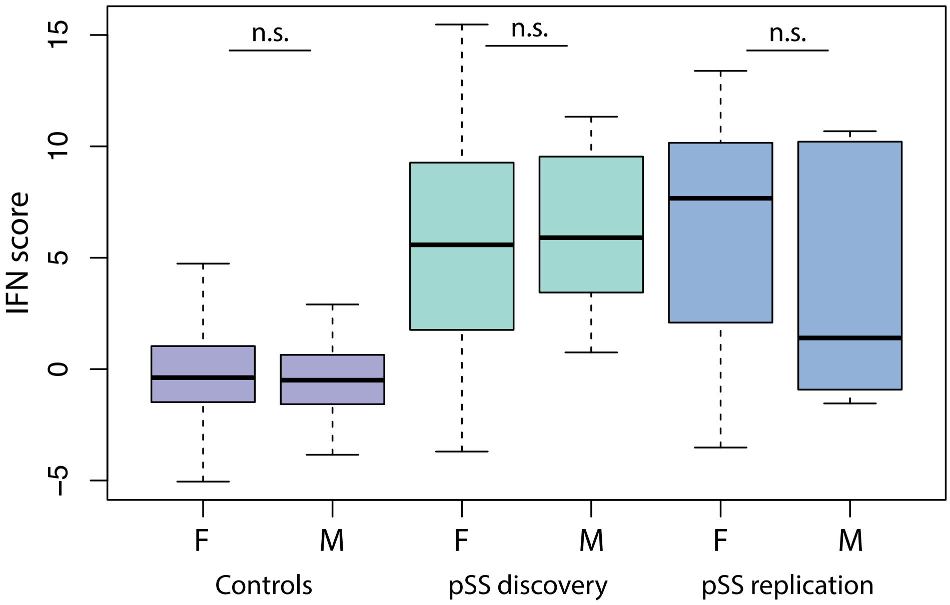

Supplementary Figure S1

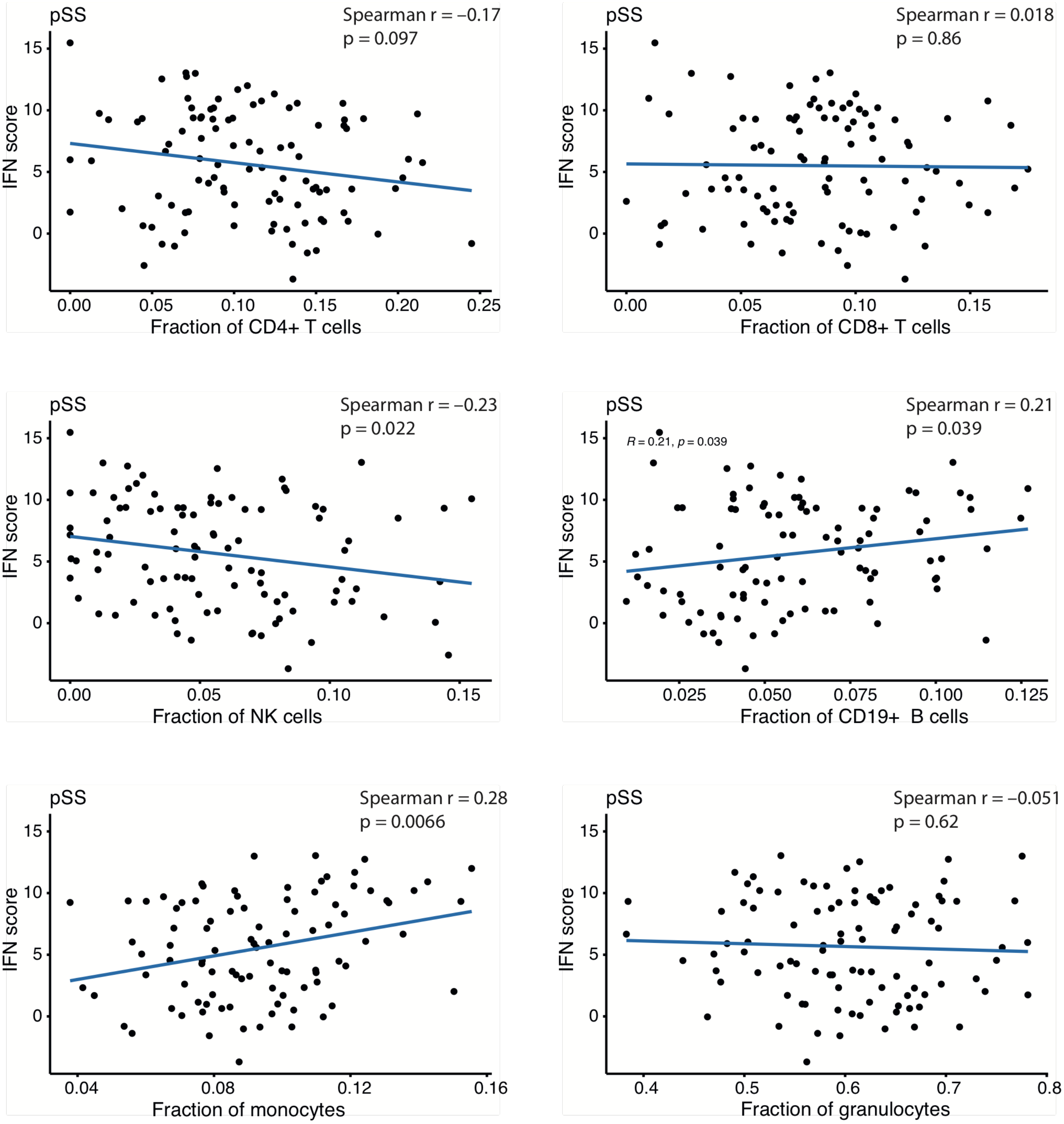

Supplementary Figure S2A

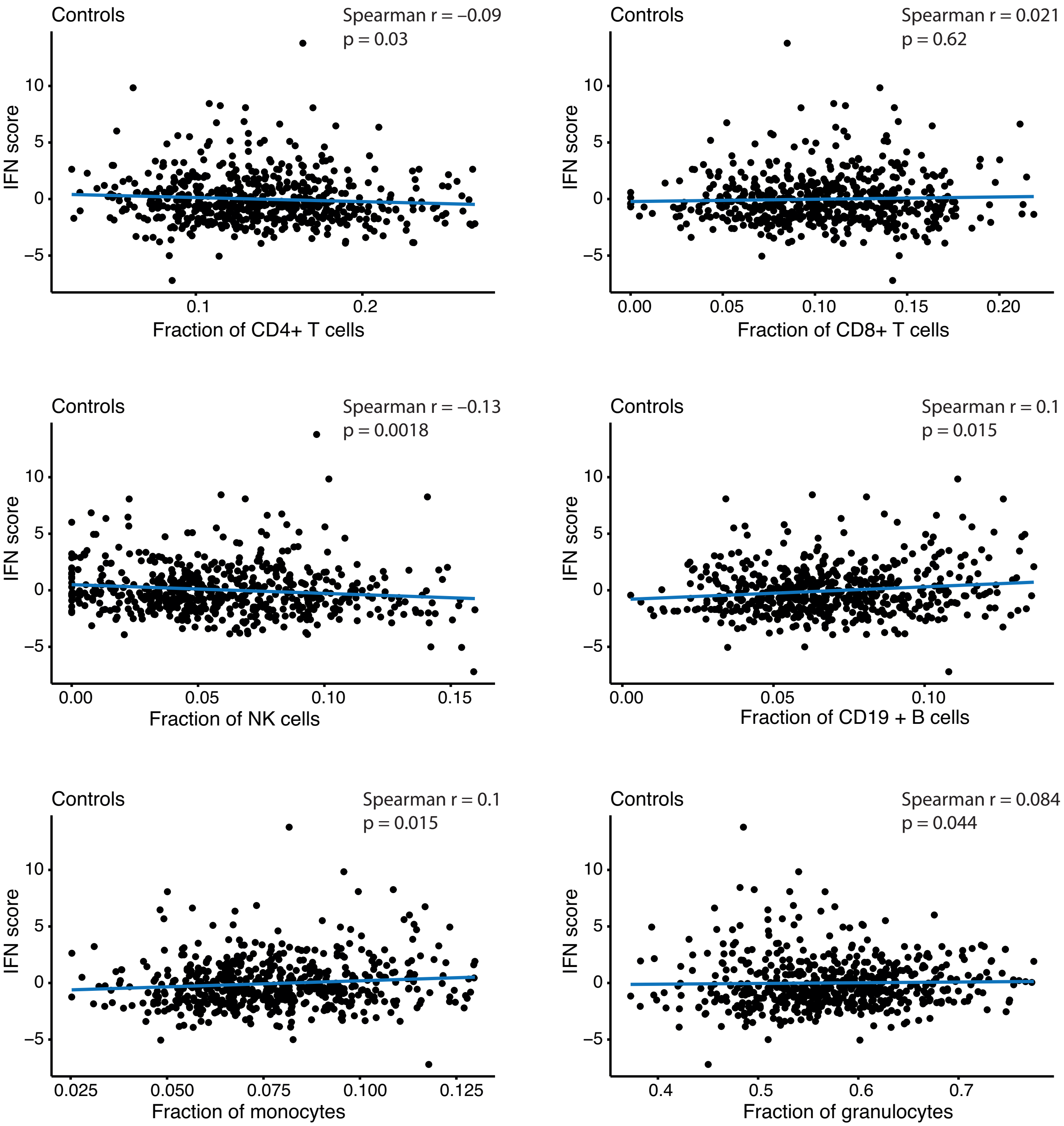

Supplementary Figure S2B

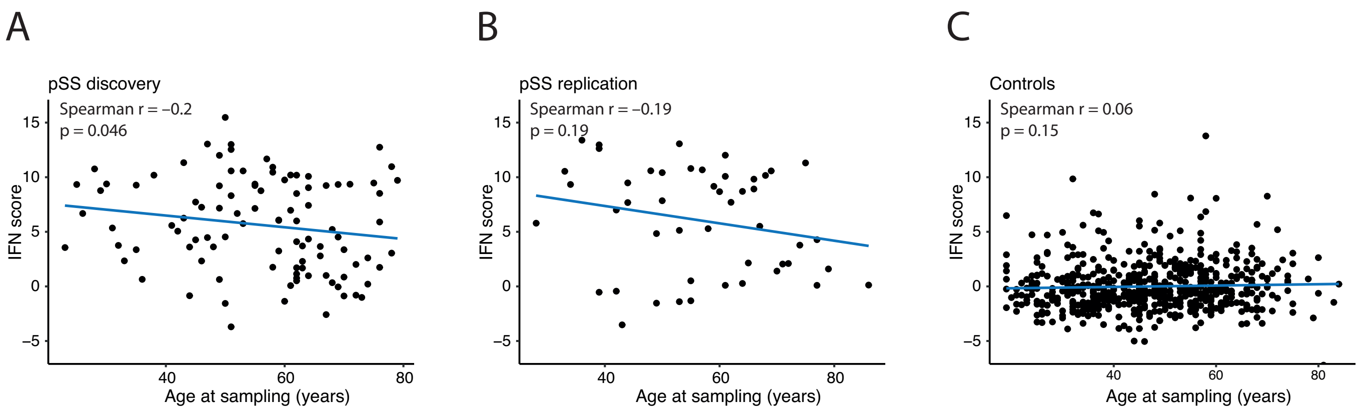

Supplementary Figure S3

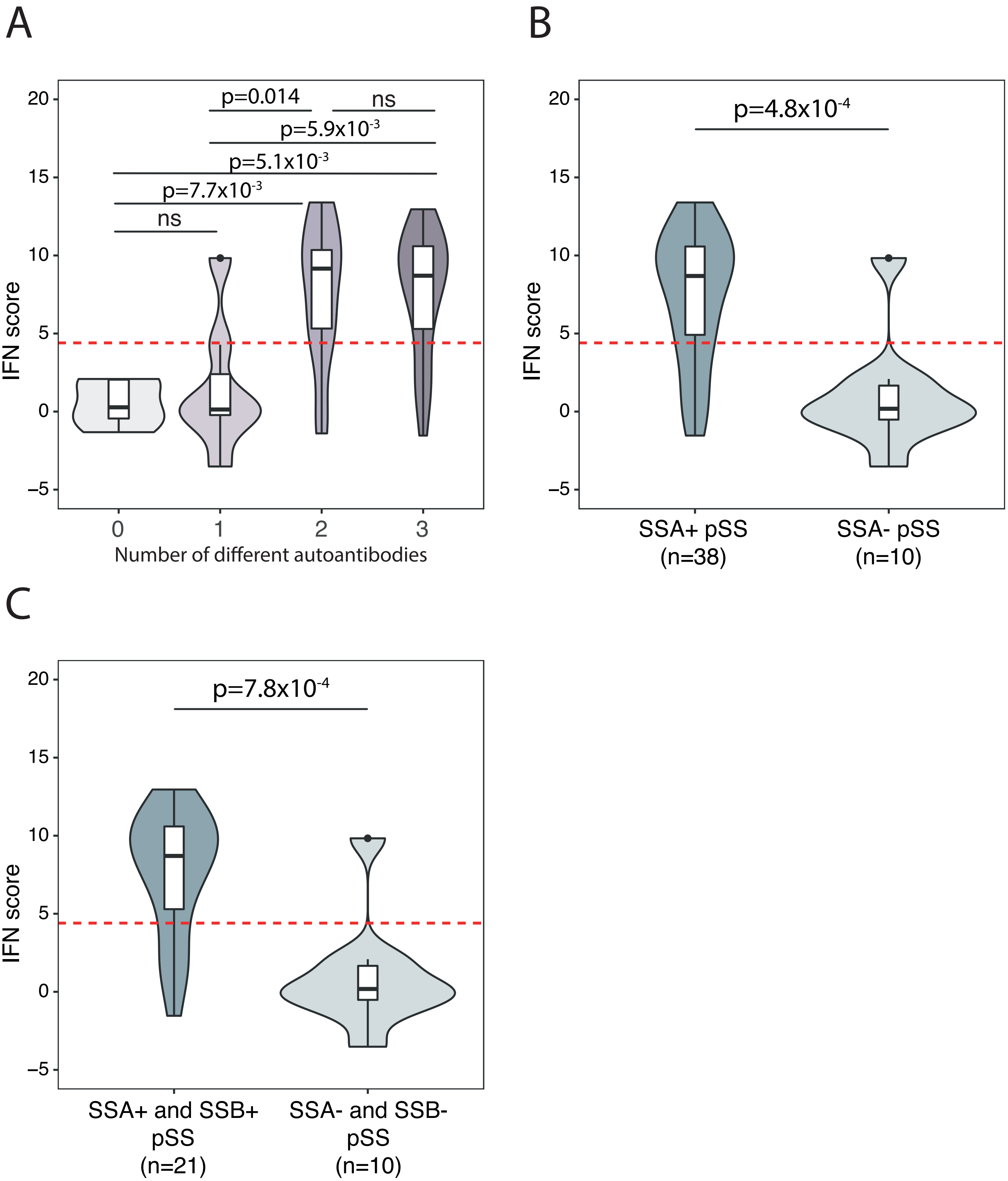

Supplementary Figure S4

Supplementary Table 1. Patients with primary Sjögren´s syndrome and lymphoma

Pat #	Cohort	Sex	Age at pSS diagnosis	SSA/SSB antibodies	Year lymphoma diagnosis	Age at lymphoma diagnosis	Year DNA sampling	Time from DNA to lymphoma (in years)	Lymphoma prior to sampling	DNAm IFN score	Lymphoma subtype	Lymphoma localisation	Treatment
1	Discovery	F	43	SSA/SSB	1988	36	2007	-19	YES	9.2	MALT	soft palate	surgery + radiation
2	Discovery	F	47	SSA/SSB	1994	49	2007	-13	YES	0.5	MALT	submandibular gland	surgery + radiation
3	Discovery	F	39	SSA/SSB	1994	49	2007	-13	YES	6.0	MALT	parotid gland	Rituximab, Leukeran
4	Discovery	F	53	SSA	1995	48	2007	-12	YES	9.8	MALT	parotid gland	surgery + radiation
5	Discovery	F	60	SSA	2008	62	2013	-5	YES	-2.6	DLBCL	lymph nodes, bone marrow	R-CHOP
6	Discovery	M	61	SSA/SSB	2002	58	2007	-5	YES	2.3	MALT	parotid gland	surgery
7	Discovery	M	43	SSA/SSB	2007	43	2007	0	NO	11.4	MALT	parotid gland	surgery + radiation
8	Discovery	F	31	SSA/SSB	2013	31	2013	0	NO	5.4	MALT	parotid glands bilateral	Rituximab + radiation
9	Discovery	M	62	NO	2010	62	2010	0	NO	0.8	Mantle cell lymphoma	tonsils bilateral	surgery
10	Discovery	F	62	NO	2001	73	2000	1	NO	2.0	High grade B-cell lymphoma NOS	ventricle, liver, lungs	CHOP
11	Discovery	F	44	SSA/SSB	2008	51	2007	1	NO	15.5	Myeloma	bone marrow, skeleton	autologous stem cell transplantation
12	Discovery	F	57	SSA	2005	65	1998	7	NO	10.9	Hodgkin	lymph nodes, bone marrow, liver	CHOP
13	Replication	F	47	SSA/SSB	2008	57	2001	7	NO	10.4	MALT	oral bucca	radiation
14	Discovery	F	73	NO	2017	82	2009	8	NO	0.2	Splenic marginal zone lymphoma	bone marrow, spleen, leukemia	Rituximab, Bendamustin
15	Discovery	F	51	SSA/SSB	2018	63	2006	12	NO	12.5	DLBCL	lymph nodes, bone marrow	R-CHOP

DLBCL: Diffuse Large B cell lymphoma; MALT: Extranodal marginal zone lymphoma of mucosa-associated lymphoid tissue; Hodgkin: Mixed cellularity classical Hodgkin lymphoma; NOS: Not otherwise specified;

CHOP: Cyclophosphamide, Hydroxydaunorubicin, Oncovine, Prednisolone; F: Female; M: Male; R-CHOP: Rituximab, Cyclophosphamide, Hydroxydaunorubicin, Oncovine, Prednisolone.
